# The Influence of Oxygen on [NiFe]–Hydrogenase Cofactor Biosynthesis and How Ligation of Carbon Monoxide Precedes Cyanation

**DOI:** 10.1371/journal.pone.0107488

**Published:** 2014-09-11

**Authors:** Sven T. Stripp, Ute Lindenstrauss, Claudia Granich, R. Gary Sawers, Basem Soboh

**Affiliations:** 1 Department of Physics, Freie Universität Berlin, Berlin, Germany; 2 Institute of Microbiology, Martin–Luther Universität Halle–Wittenberg, Halle (Saale), Germany; National Institute for Medical Research, Medical Research Council, London, United Kingdom

## Abstract

The class of [NiFe]–hydrogenases is characterized by a bimetallic cofactor comprising low–spin nickel and iron ions, the latter of which is modified with a single carbon monoxide (CO) and two cyanide (CN^−^) molecules. Generation of these ligands *in vivo* requires a complex maturation apparatus in which the HypC–HypD complex acts as a ‘construction site’ for the Fe–(CN)_2_CO portion of the cofactor. The order of addition of the CO and CN^–^ ligands determines the ultimate structure and catalytic efficiency of the cofactor; however much debate surrounds the succession of events. Here, we present an FT–IR spectroscopic analysis of HypC–HypD isolated from a hydrogenase–competent wild–type strain of *Escherichia coli*. In contrast to previously reported samples, HypC–HypD showed spectral contributions indicative of an electron–rich Fe–CO cofactor, at the same time lacking any Fe–CN^–^ signatures. This immature iron site binds external CO and undergoes oxidative damage when in contact with O_2_. Binding of CO protects the site against loss of spectral features associated with O_2_ damage. Our findings strongly suggest that CO ligation precedes cyanation *in vivo*. Furthermore, the results provide a rationale for the deleterious effects of O_2_ on *in vivo* cofactor biosynthesis.

## Introduction

Hydrogenases are ancient and widespread iron–sulfur enzymes [1]. All hydrogenases exploit low–spin transition metal ions to catalyze proton reduction and heterolytic dihydrogen oxidation following H_2_ ↔ H^+^ + H^−^ ↔ 2 H^+^ + 2 e^−^. Hydrogenases have inspired a great wealth of models [2] that exploit abundant chemical elements over noble metal catalysts like platinum. [NiFe]–, [FeFe]–, and [Fe]–hydrogenases can be distinguished based on their active site metal composition [3]. A common motif in the organometallic redox chemistry of [NiFe]– and [FeFe]–hydrogenases is a modification of the active site cofactor with carbon monoxide (CO) and cyanide (CN^−^). These are found coordinated exclusively with the iron ions and allow for efficient hydrogen turnover at minimal overpotential [4]. Generation of CO and CN^−^ *in vivo* is associated with reactive intermediates, thus evolution has established complex maturation pathways to synthesize and coordinate these diatomic ligands [5]. The catalytic cofactor of [NiFe]–hydrogenases consists of a nickel ion and an iron ion. Four cysteinyl residues coordinate the nickel site, which typically adopts the Ni^+3^ oxidation state in the catalytically inactive, oxidized ‘Ni–A’ and ‘Ni–B’ species [6], [7]. Carbon monoxide and O_2_ inhibit [NiFe]–hydrogenase activity although there are prominent exceptions [8]–[10]. Two of the aforementioned cysteine residues coordinate not only nickel but the iron ion as well. All [NiFe]–hydrogenase have a precise 2∶1 ratio of CN^–^ to CO [11] yet it is completely unclear how this is maintained. The sixth coordination site in the iron ion is shared with nickel and was found to be occupied by hydrogen and oxygen species [3], [6], [7]. We refer to the Ni–Fe cofactor as ‘[NiFe]–(CN)_2_CO’ in the following.

**Figure 1:**
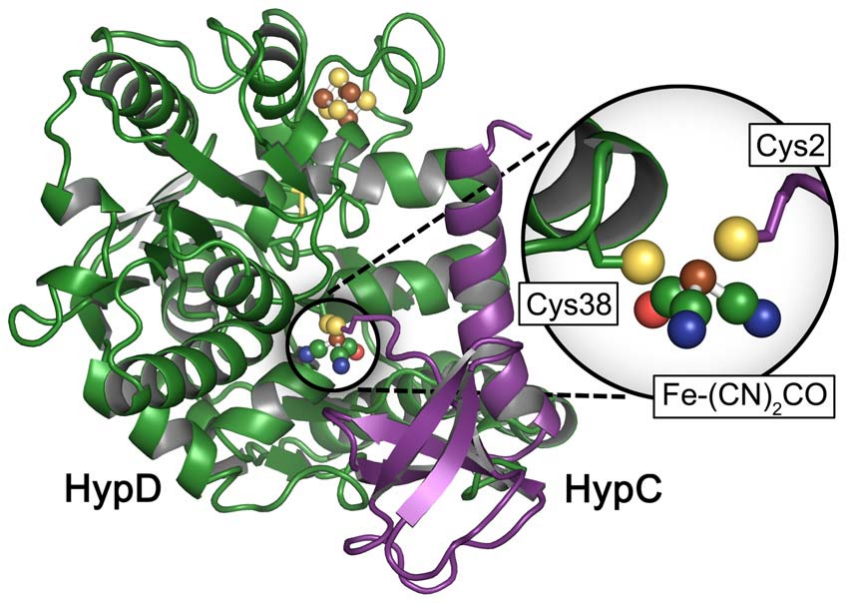
Mixed crystal structure/ homology model of the HypCD complex. The model consists of a *R. leguminosarum* HypC homology structure superimposed onto the crystal structure of HypCD from *T. kodakarensis* [20]. Fe–(CN)_2_CO was added to the N–terminus of HypC (Cys2) with the iron ion in close proximity to Cys38 of HypD and optimized in an aqueous environment. The Fe–(CN)_2_CO cofactor has been added to the model *in silico* [19].

*In vivo* generation of the [NiFe]–(CN)_2_CO cofactor (‘maturation’) has been the subject of intensive research [3], [5], [12], [13]. Minimally six accessory proteins are required specifically for cofactor biosynthesis [11], including HypA through HypF. The iron ion is modified with the three diatomic ligands prior to translocation onto the hydrogenase large subunit apo–protein. The natural source of iron has not yet been clearly identified; however, metallo–chaperone activity of HypC suggests that the small OB–fold protein is involved in iron acquisition. Furthermore, IR signatures of bound CO_2_ were detected and shown to be lost upon specific release of iron from the protein [14]. HypC forms a tight complex with HypD [15], [16], a redox active iron–sulfur protein that is the central construction site of the Fe–(CN)_2_CO moiety of the cofactor. Although no crystal structure of HypD with an intact iron site has been resolved, FT–IR analysis clearly proves the existence of the modified iron ion [17], [18]. Figure 1 shows a schematic representation of the HypC–HypD (HypCD) complex carrying the mature metal site as calculated by Albareda and co–workers [19]. The model consists of a *R. leguminosarum* HypC homology structure superimposed onto the crystal structure of HypCD from *T. kodakarensis* [20]. Fe–(CN)_2_CO was added to the N–terminus of HypC (Cys2) with the iron ion in close proximity to Cys38 of HypD and optimized in an aqueous environment [19].

While CO_2_ is a promising candidate as precursor of the CO ligand on HypD [14], the role of HypE and HypF in generation of the CN^−^ ligands has been unequivocally demonstrated [21], [22]. HypF catalyzes an ATP–dependent activation of carbamoylphosphate to carbamoyladenylate. This carbamoyl moiety is transferred to the C–terminal cysteinyl residue of HypE, yielding a thiocarbamate, which is dehydrated to thiocyanate at the expense of another ATP hydrolysis step. The crystal structure of a HypCDE complex suggests that thiocyanate from HypE is transferred to the iron site on HypD via substrate channeling [15], [23]. The order of addition of the diatomic ligands still needs to be resolved.

In this study, we isolated a mixed–state sample of HypCD from the *E. coli* K–12 wild–type strain MC4100. The protein preparation exhibits more complex infrared signatures than previously analyzed HypCD samples from hydrogenase–inactive strains [14], [17], [18], [24]. Gas treatments rendered specific changes as probed by attenuated total reflection (ATR) Fourier–transform infrared (FT–IR) absorption spectroscopy. The simultaneously extant populations reacted differently to O_2_ and CO, identifying a sample fraction devoid of iron–cyanide contributions. The IR analysis presented allows us to propose an order of events for the *in vivo* modification of the iron site on HypD. Furthermore, it explains how maturation is inhibited by O_2_.

## Materials and Methods

The strains used in this study were *E. coli* MC4100 [25] and BL21(DE3) [26]. Plasmid pT–hypDEFC_Strep_ [16] was used as the source of HypC_Strep_–HypD (HypCD in the following). For overproduction of HypCD cells were grown in 3 l of modified TB medium [18] with 100 g l^–1^ ampicillin in a 5 l conical flasks on rotary shakers at 37°C with slow rotation until an OD of 0.4 at 600 nm was reached. This ensured that the cultures were strongly O_2_–limited. At that point gene expression was induced by addition of 0.2 mg l^−1^ anhydrotetracycline and the culture was incubated at 30°C for a further 3 to 5 h up to OD_600_ = 1.0. Cells were harvested by centrifugation at 4°C at 15 000 *g* for 20 min and resuspended anaerobically at a ratio of 1 g per 3 ml buffer W (100 mM Tris–HCl, 150 mM NaCl, pH 8.0) including 2 mM sodium dithionite, 5 g ml^−1^ DNase, and 0.2 mM PMSF. Cells were disrupted by sonication (30 W for 5 min with 0.5 s pulses). Unbroken cells and debris were removed by centrifugation for 30 min at 50 000 *g* at 4°C. The supernatant derived from 10 g wet weight of cells was used for anaerobic purification of the HypCD complex. Varying sub–stoichiometric amounts of HypE were associated with the 1∶1 HypCD complex and in the interest of clarity we will henceforth refer to the complex as HypCD. Proteins were isolated using a 5 ml gravity–flow Streptactin sepharose column (IBA, Göttingen, Germany). Unbound proteins were removed by washing with five volumes of buffer W. Recombinant HypCD was eluted with buffer W including 5 mM desthiobiotin. Afterward the elutant was removed by passage through a series of Hi–Prep PD10 desalting columns (GE Healthcare). Proteins were concentrated by centrifugation at 7 500 *g* using centrifugal filters (Amicon Ultra 50 K, Millipore, Eschborn, Germany).

Fourier–transform IR spectroscopy was conducted on a rapid scan Tensor27 (Bruker Optik, Ettlingen, Germany) equipped with a three–reflection silicon crystal ATR cell (Smith Detection, Warrington, USA) as described earlier [24]. The spectrometer is situated in an anaerobic gas chamber (Coy Laboratories, Grass Lake, USA) with a N_2_/ H_2_ gas composition of 99∶1 and O_2_ levels below 0.2 ppm. Nitrogen is provided with 99.995% purity from a N_2_ generator (Inmatec, Herrsching, Germany). Carbon monoxide (^12^CO) was purchased from Linde Gas (Unterschleißheim, Germany). Isotopically labelled ^13^CO (99% ^13^CO with <5% ^18^O) was provided by Sigma Aldrich.

Gas treatments of HypCD were performed on humid films. First, a 1 µl drop of concentrated sample (∼30 g/l HypCD) was placed on top of the ATR silicon crystal and dried under water–free N_2_ to judge the amount of protein and integrity of the active site cofactor. Absolute spectra consist of 2.000 scans and are subtracted for liquid water contribution by spline functions. The crystal was covered by a gas–tight, three–way acrylic glass cell that allows for gas treatment without contamination of the anaerobic atmosphere. Second, dry N_2_ was passed through distilled water. The aerosol was fed to the ATR gas cell in order to temper the protein film and make it available for further gas treatments. Nitrogen was replaced by air or CO to induce changes in the HypCD spectrum. Eventually, difference spectra (minimally 10.000 scans) were recorded on dry films. Nitrogen was re–routed from water to a 1% (v/v) solution of H_2_O_2_ to trigger the oxidation process as reported earlier [24].

## Results

**Figure 2:**
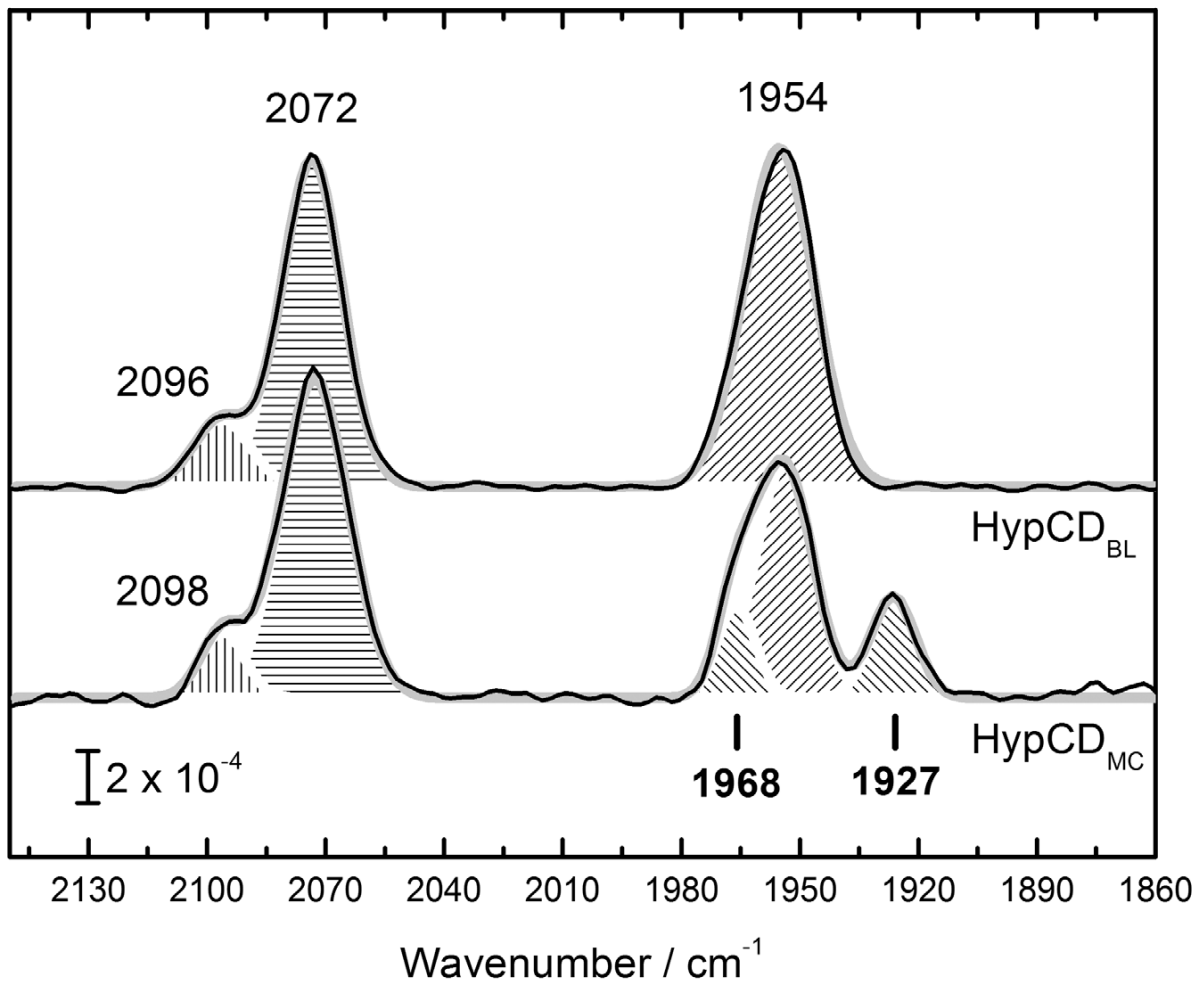
Comparison of the IR spectra of HypCD from E. coli strains BL21(DE3) and MC 4100. HypCD_BL_ shows the characteristic signature [17], [18] of one CO (1954 cm^−1^, diagonal hatch) and two CN^−^ ligands (2072, 2096 cm^−1^, straight hatch). HypCD_MC_ has a novel contribution in the CO region (1927 cm^−1^) and shows a shoulder blue–shifted to the CO peak at 1954 cm^−1^. The latter shoulder fits best to a Gaussian peak with a center wavenumber of 1968 cm^−1^. The bold gray line in the background represents an overall fit of the spectrum.

The HypCD protein complex was over–produced and purified anaerobically via Streptactin sepharose affinity chromatography from extracts of *E. coli* MC4100 (referred to as HypCD_MC_) by making use of the C–terminal strep–tag on HypC. Strain MC4100 typically synthesizes three [NiFe]–hydrogenases after anaerobic growth and has the full complement of accessory proteins (HypABCDEF) required for maturation [25], [27]. Figure 2 shows FT–IR spectra comparing HypCD_MC_ and HypCD_BL_ isolated from *E. coli* strain BL21(DE3). This strain is defective in synthesis of active hydrogenases [28] and was transformed with plasmid phypCDEF to overproduce the accessory proteins. HypCD_BL_ exhibits contributions from two bands at 2072 and 2096 cm^−1^ and a single contribution at 1954 cm^−1^. The latter signal has been attributed to Fe(II)–CO, while both former bands stem from Fe(II)–CN1 and Fe(II)–CN2 [17], [18]. In the spectrum of HypCD_MC_, three bands fit with 2072, 2098, and 1954 cm^−1^, corresponding to Fe(II)–CN1, Fe(II)–CN2, and Fe(II)–CO, respectively. A new peak appeared at lower wavenumbers with a maximum at 1927 cm^−1^. The main Fe(II)–CO peak shows a clear shoulder at higher wavenumbers, fitting best with 1968 cm^−1^.

**Figure 3:**
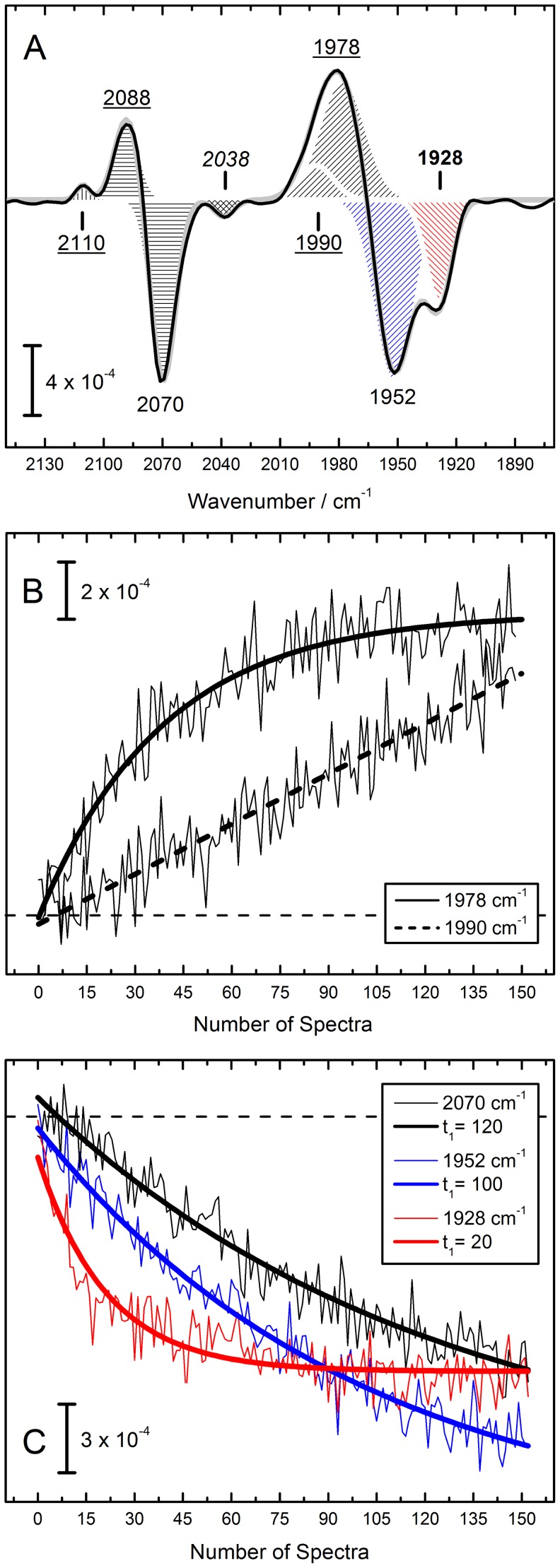
H_2_O_2_ IR difference spectrum of HypCD_MC_. (Fig. 3A) Negative features represent the as–isolated state (1928 and 1952 cm^−1^ for CO in red and blue hatch, respectively, and 2070 cm^−1^ for CN^−^), positive features are attributed to the oxidized, instable Fe(III)–(CN)_2_CO state with 1978/1992 cm^−1^ attributable to CO and 2088 and 2110 cm^−1^ attributable to the CN^−^ ligands [24]. A new band arises at 2038 cm^−1^ (cross hatch). (Fig. 3B) While the contribution at 1978 cm^−1^ exponentially increases after contact of the sample with H_2_O_2_, the band at 1990 cm^−1^ obeys a rather linear kinetic behavior. (Fig. 3C) The previously identified contributions at 1952 and 2070 cm^−1^ vanish simultaneously [24], however, the peak at 1928 cm^−1^ showcases a four to five times faster absorption decay. All kinetics are plotted in ‘Number of Spectra’ to avoid misinterpretation of temporal information.

HypCD_BL_ has been shown previously to react with H_2_O_2_ [24], with rapid oxidation of the iron cofactor due to Fenton chemistry [29], [30]. We performed a kinetic analysis of the effect of H_2_O_2_ on HypCD_MC_ to identify coupled vibrations. Figure 3A shows the final difference spectrum of HypCD_MC_ subjected to H_2_O_2_ oxidation. Negative peaks represent the state before the treatment. Strong bands appear at 1928, 1952, and 2070 cm^−1^. One negative band fits to 2038 cm^−1^. Peaks at 1968 and 2096 cm^−1^ are not visible because they are masked by large positive contributions at 1978/1990 cm^−1^ and 2088/2110 cm^−1^. While the peaks at 2088/2110 cm^−1^ can readily be assigned to Fe(III)–CN1 and CN2 [24], the 1978/1990 cm^−1^ peaks were identified based on their deviating kinetic behavior in the oxidation process.

Figure 3B shows the increase of the 1978 and 1990 cm^−1^ contribution over time. In an earlier publication we followed oxidation from Fe(II)–CO to Fe(III)–CO as a result of H_2_O_2_ treatment via the 1990 cm^−1^ band [24]. However, upon closer examination it seems the initial product of oxidation is a band that fits best to 1978 cm^−1^ and whose kinetics can be monitored exponentially. Underlying this is a somewhat linear process that includes a non–redox shift to 1990 cm^−1^. In the current experiment this process was incomplete, which is why in fig. 3A a two–component Gaussian fit in the region of 1978/1990 cm^−1^ was used. The 1928 cm^−1^ peak was not found to be coupled with the 1952 and 2070 cm^−1^ contributions. Figure 3C depicts the diverging reaction kinetics. The new band at 1928 cm^−1^ (red slope) decreased four to five times faster than the Fe(II)–CO and Fe(II)–CN1 marker bands (blue and black slope, respectively). This hints at a mixed population in HypCD_MC_ in contrast to the homogenous isolation that has been published for HypCD_BL_ [17], [18].

**Figure 4:**
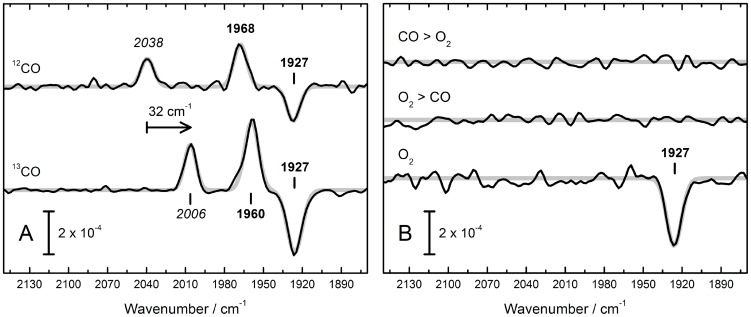
IR difference spectra of HypCD_MC_ that show the effect of ^12^CO, ^13^CO, and O_2_. (Fig. 4A) Binding of external ^12^CO (^13^CO) produces a new peak, 2038 cm^−1^ (2006 cm^−1^) and induces a shift of the original Fe–CO peak from 1927 to 1968 cm^−1^ (1960 cm^−1^). Note these spectra do not give rise to difference signals in the CN^−^ region. (Fig. 4B) A strong negative contribution at 1927 cm^−1^ is observed. Double difference spectra depict on the influence of O_2_ on CO–soaked sample (CO>O_2_) and *vice versa* (O_2_>CO). No additional difference bands are observed. Note these spectra do not give rise to difference signals in the CN^−^ region.

Semi–dry films of HypCD were treated with air (referred to as O_2_ henceforth), ^12^CO, and ^13^CO. While HypCD_BL_ was insensitive to either O_2_ or CO, HypCD_MC_ exhibited distinct changes. Figures 4A and B show the resulting difference spectra. Only the band at 1927 cm^−1^ reacted with CO and O_2_. Upon incubation with ^12^CO gas, a shift of 1927 to 1968 cm^−1^ was recorded (fig. 4A). Simultaneously, a pronounced peak at 2038 cm^−1^ appeared. A peak at 2038 cm^−1^ was observed earlier as a negative contribution in the difference spectrum of HypCD_MC_ after H_2_O_2_ oxidation (fig. 3A). The 1968 cm^−1^ band was assigned as a contribution to the broad 1954 cm^−1^ peak of HypCD_MC_ in fig. 2. In contrast, the peak centered at 2038 cm^−1^ was absent from the absolute spectrum. Binding of ^13^CO to HypCD gave rise to the expected isotope shift. A new peak appeared at 2006 cm^−1^, and the shift of 1927 to 1960 cm^−1^ was eight wavenumbers smaller than for the reaction with ^12^CO. A single negative peak appeared at 1927 cm^−1^ upon treatment with O_2_ (fig. 4B). This indicates a loss of absorbing molecules. Oxygen was not found to react with HypCD_MC_ after CO treatment, and *vice versa*. It is important to note that no difference signals were observed for the peaks assigned to Fe(II)–CO (1954 cm^−1^), Fe(II)–CN1 (2072 cm^−1^), and Fe(II)–CN2 (2098 cm^−1^). Small changes in the cyanide region became visible only upon prolonged incubation under 1 mbar ^13^CO (see fig. S1). This effect was not observed with ^12^CO.

## Discussion

Synthesis of CN^−^ from carbamoyl–phosphate for the active site cofactor of [NiFe]–hydrogenases has been elucidated in ever–increasing detail since Reissmann and co–workers published their pioneering work in 2003 [21]. Crystal structures of the accessory proteins HypE and HypF have been resolved, both individually and in the relevant quaternary complexes, such as CDE [15], [20], [31], [32]. Recently, a specifically cyanated form of HypE has been crystallized after incubation with OCN^−^ and ATP [23]. Moreover, the HypD enzyme was characterized as a central scaffold in [NiFe]–hydrogenase maturation [17], [18]. HypD binds a single iron ion as acceptor of the CN^−^ ligands from cyanated HypE *in vivo* [16]. HypD carries a [4Fe–4S] cluster linked to an ‘active site’ iron ion within tunneling distance via one or two disulfide bridges [20]. HypD is discussed to be a redox enzyme that catalyzes the reductive transfer of CN^−^ and CO to iron in an ATP–dependent reaction. The metabolic origin of CO is matter of ongoing debate [3] although prominent IR signatures on HypC and HypCD hint at iron–bound CO_2_ as a precursor of the CO ligand [14], [18], [24]. While it is reasonably clear what the source of the CN^−^ ligands is, and strong circumstantial evidence for one possible source of CO has been suggested, precise details of how the 2∶1 ratio of CN^−^ to CO is maintained remains unclear.

Isolation of HypCD from *E. coli* BL21(DE3) phypCDEF revealed an iron cofactor with two CN^−^ ligands (CN1 at 2072 and CN2 at 2098 cm^−1^) and a single CO ligand (1955 cm^−1^). Strain BL21(DE3) naturally lacks hydrogenase activity because of deficiencies in nickel uptake and insertion [28], which presumably results in accumulation of the HypCD complex as the final acceptor of a pre–formed Fe–(CN)_2_CO cofactor [17], [18]. In this study, we isolated a mixed–state sample of HypCD from the hydrogenase–active *E. coli* wild–type strain MC4100. The increased spectral features probably result from the fact that hydrogenase maturation is unrestricted when compared with BL21(DE3). HypCD_MC_ revealed an additional band at 1927 cm^−1^ and a shoulder of the main peak at 1954 cm^−1^, which fits best with 1968 cm^−1^. It is unlikely for this new state to represent an ‘off–pathway’ intermediate as trace amounts of the 1927 cm^−1^ contribution have been observed previously in difference spectra of a preparation derived from BL21(DE3) [24]; however this contribution barely accumulated in the hydrogenase–negative strain. The signal was attributed to a reduced population Fe(I)–CO, well in accord with a shift of ∼30 cm^−1^. In [NiFe]–hydrogenases, a band at 1922 cm^−1^ was assigned to the reduced, hydride–binding ‘SI_R1_’ state [33]. The reduced H–cluster of [FeFe]–hydrogenases includes a CO ligand that absorbs at 1916 cm^−1^ (Fe_d_(I)–CO) [34]. Therefore, we assign the 1927 cm^−1^ band to Fe(I)–CO. We rule out the possibility of a second CO ligand to Fe(II)–(CN)_2_CO because the positions of the three original ligands are unchanged; an additional ligand on the central coordination metal site would result in a blue–shift of the spectral signature of the whole cofactor. No peaks indicative of a conceivable Fe(I)–CN^−^ moiety were detected. However, these contributions might be lost in the combination band of H_2_O that partially masks iron–cyanide absorption.

Further support for this finding was yielded by a kinetic analysis of the effects of H_2_O_2_ treatment on the Fe–(CN)_2_CO cofactor. We have shown previously that H_2_O_2_ rapidly oxidizes the iron ion from Fe(II) to Fe(III) by Fenton chemistry [24]. While the overall effect on HypCD_MC_ is not significantly different to what was reported for HypCD_BL_, the Fe(I)–CO (1927 cm^−1^) signature was found to react four to five times faster to chemical oxidation, thus being uncoupled from the other spectral contributions. The three diatomic ligands bound to Fe(II) shift with basically identical time constants. This comparison provides an explanation how the HypCD_MC_ spectrum is a mixture of (at least) two redox states.

The kinetic analysis elucidated two further important findings. First, the initial product of H_2_O_2_ oxidation of Fe(II)–CO is not Fe(III)–CO at 1990 cm^−1^ as previously suggested [24] but rather results in a band at 1978 cm^−1^ (+24 cm^−1^). The latter peak forms first but shifts to 1990 cm^−1^ in a non–redox process fitting to a linear behavior. Radical chemistry associated with H_2_O_2_ oxidation, plus the instability of a Fe(III)–CO is very likely to cause structural changes in the active site environment. Infrared signatures of gaseous CO_2_ upon prolonged H_2_O_2_ incubation results from highly oxidized protein. The second finding is that the reduced–*versus*–oxidized difference spectrum revealed a (negative) contribution at 2038 cm^−1^ not visible in the absolute spectrum of HypCD_MC_, possibly due to a contribution of liquid water (which negate each other in the difference spectrum). We were able to assign this peak only after analysis of the effect of O_2_ and CO on HypCD_MC_.

The O_2_ sensitivity of many [NiFe]– and all [FeFe]–hydrogenases is well documented [8]–[10], [35]–[37]. It is also known that O_2_ not only interferes with active hydrogenases but inhibits cofactor maturation as well [5]. In order to gain comprehensive knowledge of O_2_ inhibition *in vivo* it is important to understand at which point in maturation the oxidant competes with cofactor ligation. Remarkably, the pre–formed Fe(II)–(CN)_2_CO moiety on HypCD and on HypD was found not to react with O_2_. We could show previously that HypD alone is able to coordinate the Fe–(CN)_2_CO cofactor [24]. Next to the three non–protein ligands we experimentally identified a cysteine residue as occupying one binding site of low–spin Fe(II) [18], [24]. Obviously, the non–reactivity of HypCD_BL_ towards O_2_ and CO requires complete saturation of all binding sites.

Oxygen treatment of the HypCD_MC_ mixed–state sample results in a loss of the 1927 cm^−1^ peak exclusively. No shift was detected in the other bands and we could not identify any new bands. The results strongly suggest that this 1927 cm^−1^ contribution (potentially a precursor Fe(I)–CO form of the cofactor) is released from HypCD in the presence of O_2_. Interestingly, when the HypCD_MC_ film is treated with CO prior to contact with O_2_, no such loss is observed. It is tempting to suggest a protective CO inhibition against O_2_ damage, as it has been observed for [FeFe]–hydrogenases [36]. Carbon monoxide was not found to react with O_2_–soaked films, which argues in favor of a displacement of the Fe(I)–CO site upon incubation with O_2_.

The effect of CO on HypCD_MC_ is even more illustrative. The band at 1927 cm^−1^ shifts to 1968 cm^−1^ and a new peak forms at 2038 cm^−1^. We interpret the latter peak to be due to exogenous CO at Fe(I), accompanied by a +41 cm^−1^ shift of the original CO peak (Fe(I)–(CO)_2_). This is reasonable for coupled vibrations. Treatment of HypCD_MC_ with ^13^CO results in the same effect, however, the specific shifts are different and allow for definitive assignment of the 2038 cm^−1^ band to exogenous ^12^CO (2004 cm^−1^ for ^13^CO). Accordingly, the effect of ^13^CO binding to Fe(I)–CO is slightly smaller (8 cm^−1^) in comparison to ^12^CO. For both ^12^CO and ^13^CO, no changes were detected in the CN^−^ region although intensive and prolonged incubation with ^13^CO partly replaces the natural CO ligand in Fe(II)–(CN)_2_CO (ligand scrambling [34]) and gives rise to iron–cyanide difference signals (see fig. S1). Re–examination of the absolute spectrum of HypCD_MC_ reveals that both the 1968 cm^−1^ and 1927 cm^−1^ bands arise from the mixed–state sample. Obviously, HypCD_MC_ includes not only an electron–rich intermediate Fe(I)–CO but a CO–inhibited species Fe(I)–(CO)_2_ as well. Self–poisoning of hydrogenase samples by CO is a frequent phenomenon referred to as ‘cannibalization’ [34] and explains why it is not surprising to find a fraction of CO–contaminated enzyme in HypCD_MC_. This interpretation is supported by the negative band at 2038 cm^−1^ in the H_2_O_2_ difference spectrum, which must have been present initially but was potentially masked by the H_2_O combination band.

## Conclusion

**Figure 5:**
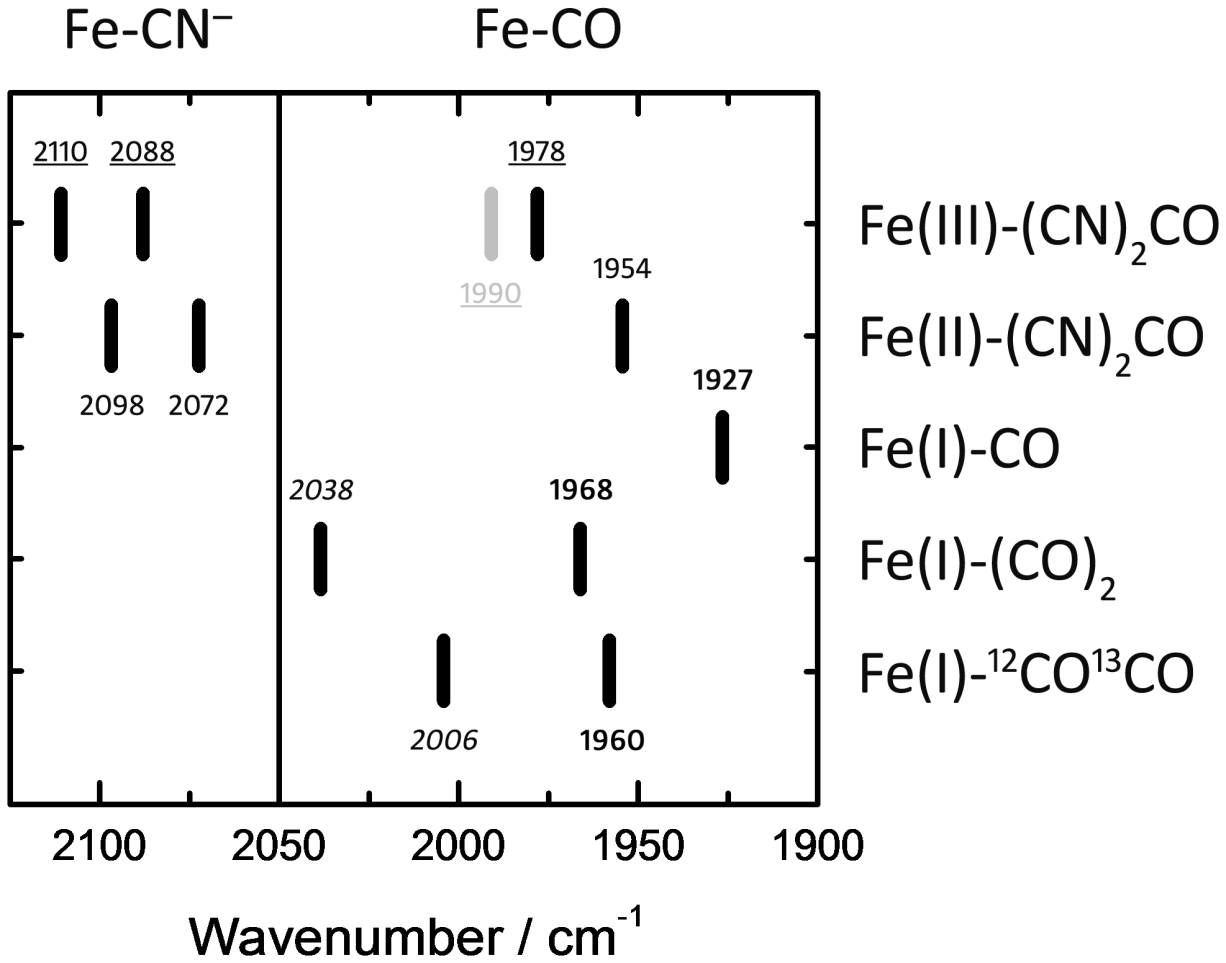
Conclusive comparison of the detected bands on HypCD_MC_. Including Fe(II)–(CN)_2_CO (as–isolated), Fe(III)–(CN)_2_CO (oxidized by H_2_O_2_), Fe(I)–CO (reduced fraction), Fe(I)–(CO)_2_, and Fe(I)–^12^CO^13^CO. See discussion for details.

The novel population isolated from the HypCD_MC_ mixed–state sample reacts with O_2_ and binds external CO. Steady–state and kinetic comparison with the characterized Fe(II)–(CN)_2_CO cofactor tentatively allows the assignment of the 1927 cm^−1^ contribution to Fe(I)–CO. Most importantly, no effect in the iron–cyanide region of the spectrum was observed upon treatment with O_2_, CO, or both. We could identify additional CO–binding species assigned to Fe(I)–(CO)_2_ (1968, 2038 cm^−1^) and Fe(I)–^12^CO^13^CO (1960, 2006 cm^−1^). No cyanide–binding intermediate was isolated, and CO_2_ was also absent from the sample. Figure 5 shows a plot of the identified HypCD_MC_ redox states. While Fe(I)–(CO)_2_ might be a ‘contamination’ due to cannibalization or to CO–scrambling, Fe(I)–CO possibly represents the first of two inter–dependent steps in cofactor synthesis: The first step involves binding of CO to an Fe(I) ion on HypD, or binding of Fe–CO_2_ delivered by HypC [14] and reduction to Fe(I)–CO by a CO_2_ reductase–like mechanism, e.g. by HypD [38]; the second step results in binding of CN1 and CN2 to Fe(I)–CO as proposed by Watanabe and co–workers [15]. Our data potentially explains how externally added CO can be incorporated into the active site during *in vivo* biosynthesis but fails to exchange with bound CO in the mature active site [39], [40]. The same observation was made for O_2_; once Fe(II)–(CN)_2_CO is formed HypCD is not affected. These findings suggest that O_2_–sensitivity occurs exclusively at an earlier stage of maturation, e.g. immediately prior to addition of the CN^–^ ligands and explains how cofactor synthesis is inhibited by O_2_ *in vivo*.

In a recent study Bürstel and co–workers have suggested that the binding of CN^−^ ligands occurs prior to CO–ligation of the iron ion [17]. However, in that publication no iron–cyanide species devoid of CO was presented. We have identified CO bound to iron that lacks any CN^−^ components, strongly suggesting that CO is the first diatomic ligand to be attached. This order of addition explains how the conserved 2∶1 ratio of CN∶CO is maintained in all [NiFe]–hydrogenases.

## Supporting Information

Figure S1 **Cyanide ligand scrambling upon prolonged incubation with ^13^CO.** At 1 mbar and incubation times +1 h (room temperature, ambient light) ^13^CO not only binds to Fe(I)–CO but continuously replaces the ‘natural’ ^12^CO ligand of the Fe(II)–(CN)_2_CO cofactor whose spectrum serves as background in fig. S1. The black trace shows ^12^CO binding to a sample of HypCD_MC_ as discussed in the main script. The red trace (^13^CO binding) gives rise to derivative–shaped signals in the Fe(II)–CN^–^ region (here 2050–2100 cm^−1^). Both spectra were recorded under comparable conditions.
(PDF)
