# Supplementary material for: The Influence of Oxygen on [NiFe]–Hydrogenase Cofactor Biosynthesis and How Ligation of Carbon Monoxide Precedes Cyanation
Source: PLoS One. 2014 Sep 11;9(9):e107488. doi: 10.1371/journal.pone.0107488 (PMC4161419; doi:10.1371/journal.pone.0107488)
Supplement: Figure S1 — Cyanide ligand scrambling upon prolonged incubation with 13CO. At 1 mbar and incubation times +1 h (room temperature, ambient light) 13CO not only binds to Fe(I)–CO but continuously replaces the ‘natural’ 12CO ligand of the Fe(II)–(CN)2CO cofactor whose spectrum serves as background in fig. S1. The black trace shows 12CO binding to a sample of HypCDMC as discussed in the main script. The red trace (13CO binding) gives rise to derivative–shaped signals in the Fe(II)–CN– region (here 2050–2100 cm−1). Both spectra were recorded under comparable conditions. (PDF) [file pone.0107488.s001.pdf]

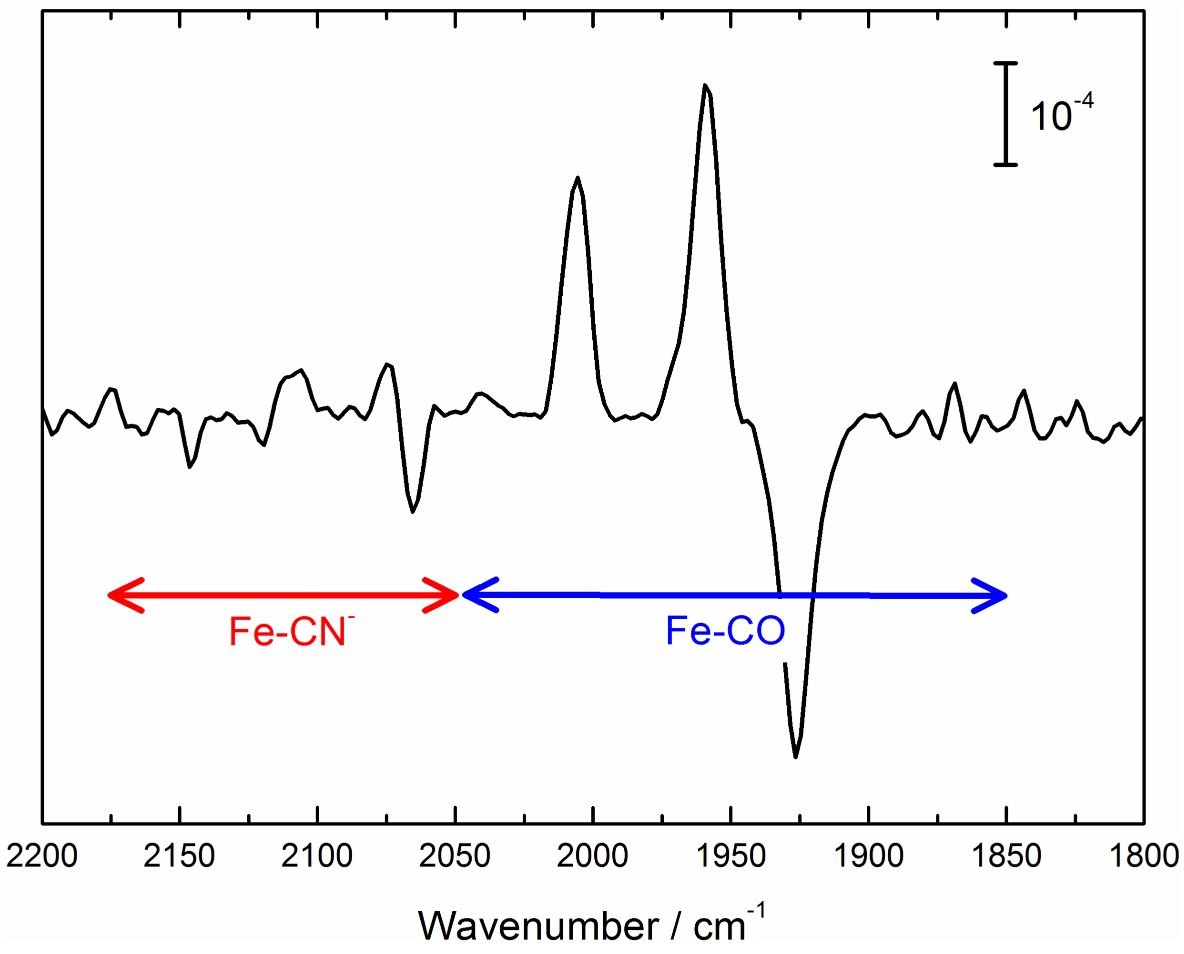

Figure S1 – Extended incubation of HypCD$_{MC}$ with ^{13}CO. After 2h at 1 mbar ^{13}CO (ambient light), some of the intrinsic ^{12}CO exchanges to ^{13}CO. Cyanide ligands are coupled to CO via the iron ion in Fe(II)-$(CN)_2$CO thus difference signals from 2150 – 2050 cm^{-1} are attributable to isotope exchange.
